# Investigating Community Pharmacy Take Home Naloxone Dispensing during COVID-19: The Impact of One Public Health Crisis on Another

**DOI:** 10.3390/pharmacy9030129

**Published:** 2021-07-23

**Authors:** George Daskalakis, Ashley Cid, Kelly Grindrod, Michael A. Beazely

**Affiliations:** School of Pharmacy, University of Waterloo, 10 Victoria St S A, Kitchener, ON N2G 1C5, Canada; George.daskalakis@uwaterloo.ca (G.D.); Ashley.cid@uwaterloo.ca (A.C.); kelly.grindrod@uwaterloo.ca (K.G.)

**Keywords:** naloxone, harm reduction, community pharmacy, pharmacy services, overdose

## Abstract

A recent report found that the number of opioid-related deaths in Ontario in the first 15 weeks of the COVID-19 pandemic was 38.2% higher than in the 15 weeks before the pandemic. Our study sought to determine if pharmacy professionals self-reported an increase or decrease in naloxone provision due to the pandemic and to identify adjustments made by pharmacy professionals to dispense naloxone during the pandemic. A total of 231 Ontario community pharmacy professionals completed an online survey. Pharmacy professionals’ barriers, facilitators, and comfort level with dispensing naloxone before and during the pandemic were identified. The sample consisted of mostly pharmacists (99.1%). Over half (51.1%) reported no change in naloxone dispensing, while 22.9% of respondents reported an increase and 24.7% a decrease. The most common adjustments made during the pandemic were training patients how to administer naloxone over video or phone, delivering naloxone kits, and pharmacy technicians offering naloxone at prescription intake. Over half (55%) of participants said the top barrier for dispensing was that patients did not request naloxone. Naloxone distribution through pharmacies could be further optimized to address the increased incidence of overdose deaths during the pandemic. Future research should investigate the reasons for changes in naloxone dispensing.

## 1. Introduction

The Coronavirus Disease 2019 (COVID-19) pandemic has had a significant impact on the opioid crisis, as seen by the increasing number of opioid-related overdoses and deaths [1]. Between January 2016 and June 2020, Canada reported more than 17,500 apparent opioid toxicity deaths [1]. During this period, the highest quarterly count occurred between April and June of 2020 [1]. In Ontario, the number of opioid-related deaths in the first 15 weeks of the COVID-19 pandemic was 38.2% higher than in the 15 weeks before the pandemic [2]. It is estimated that if that trend continued, the number of Ontario opioid-related deaths in 2020 would be 2271, representing a 50% increase compared to 2019 [2] and a 56.6% increase from 2018 [3]. Pharmacists are highly accessible healthcare professionals and play a significant role in reducing the effects of the opioid crisis by offering naloxone kits. An Ontario population-based study showed that between 1 July 2016 and 31 March 2018, Ontario community pharmacies dispensed 91,069 naloxone kits to 67,910 unique individuals [4]. 

According to the World Health Organization (WHO), approximately 115,000 people died of an opioid overdose in 2017, and the number of opioid overdoses has increased in in many countries in recent years [5]. For example, between 2010 and 2018, the number of individuals who died from an opioid overdose increased by 120% in the United States [5]. The CDC estimates the cost incurred in 2017 for fatal opioid overdoses and opioid use disorder in the United States to be 1.02 trillion [6]. 

With the COVID-19 pandemic, the typical methods of dispensing medications (including naloxone) have experienced alterations as a result of physical distancing guidelines. Private communications with corporate pharmacies suggest that, within their operations, naloxone dispensing rates have decreased since the beginning of the COVID-19 pandemic. In addition to the changes in the provision of naloxone to the public, access to various addiction and other support services has also been affected [7]. For example, the Ontario Ministry of Health has acknowledged that it may be more challenging for some to access publicly funded mental health and addictions providers or peer support groups [8]. With the physical distancing measures in place, there are concerns that isolation, anxiety, and financial stress may negatively impact people with substance use disorders or those at risk of developing one [9]. Additionally, physical distancing measures may increase the likelihood of an individual using opioids alone [7]. This prompted the Chief Public Health Officer of Canada to release a statement in which they reiterated the importance of not using drugs alone and always carrying naloxone when using drugs [7]. 

In recent years, there have been multiple articles published on the provision of naloxone by pharmacists. In fact, there have been several scoping and systematic reviews analyzing the topic from various perspectives [10,11,12,13,14]. A 2021 scoping review by Cid et al. identified 19 surveys that evaluated naloxone provision by pharmacy professionals [14]. These surveys evaluated naloxone provision in terms of dispensing frequency, naloxone availability, pharmacy staff knowledge, barriers to provision, facilitators to provision, and pharmacy professionals’ attitudes toward naloxone [14]. For example, an online survey by Carpenter et al. identified barriers to naloxone dispensing as time, inadequate pharmacist training, and the perception that patients do not understand the purpose of naloxone [15]. Facilitators identified included pharmacists feeling more comfortable discussing naloxone and naloxone training for pharmacists [15]. The scoping review by Cid et al. also identified 10 studies demonstrating a disparity in naloxone access in independent and rural pharmacies [14]. These pharmacies were less likely to stock and dispense naloxone compared to their urban and chain pharmacy counterparts [14]. 

It is important to determine how the COVID-19 pandemic has affected naloxone provision by community pharmacies, and no previous studies have been conducted in this area. Given that the opioid crisis appears to be worsening as the COVID-19 pandemic progresses [1,2], it is critical that we develop an understanding of how the public’s access to naloxone via community pharmacies may be affected and what adjustments these professionals are or are not making to distribute naloxone. A survey is required to develop a stronger understanding of the issue and will allow us to identify methods of enhancing naloxone provision in the community pharmacy setting. This study will allow for learning about adjustments being made to overcome potential barriers which may help strengthen future pharmacy naloxone programs, even once the global pandemic ends. With that in mind, the following research question was formulated: how has the COVID-19 pandemic affected the naloxone dispensing habits of Ontario community pharmacy professionals? The primary objective of this survey was to determine if naloxone provision by Ontario pharmacy professionals has increased or decreased as a result of the COVID-19 pandemic. A secondary objective was to identify adjustments made by pharmacy professionals to dispense naloxone during the COVID-19 pandemic.

## 2. Materials and Methods

This survey was conducted using the online questionnaire software, Qualtrics (www.qualtrics.com, Utah, UT, USA, January–February 2021). The survey consisted of demographic questions and questions related to the provision of naloxone. There was a mix of both open-ended and closed-ended questions. Eligible participants needed to be a licensed pharmacy technician, or a Part A pharmacist registered with the Ontario College of Pharmacists, working in a community pharmacy setting for the past year. While pharmacists evaluate clinical and technical appropriateness of all prescriptions, registered pharmacy technicians can independently verify the technical component of prescriptions, leaving any clinical component for verification by a Part A pharmacist [16]. In Ontario, a Part A pharmacist is a pharmacist who provides direct patient care and works a minimum number of hours in a patient care setting [17]. The community pharmacy setting was defined in the survey as a corporate, franchise, banner, or independently-owned pharmacy. Participants were screened in Qualtrics for eligibility prior to accessing the survey (Appendix A). The survey questions were developed by two researchers (A.C. and G.D.) and subsequently underwent revision by 10 fourth year pharmacy students and four School of Pharmacy faculty members (including K.G. and M.A.B.).

The survey was open from 22 January 2021 to 17 February 2021. It was distributed to potential participants in two ways. First, A.C. and G.D. advertised the survey twice through Twitter, LinkedIn, and Facebook groups consisting primarily of Ontario pharmacy professionals. Second, using the Ontario College of Pharmacists’ database, an email with a link to the survey was distributed on three occasions to all actively licensed community pharmacy professionals who had previously given the College permission to share their email for research purposes.

The demographic questions (Appendix B) collected the following information: professional title, educational background, years in practice, and the type of community pharmacy participants primarily practice in. Additionally, participants were asked to indicate the population size of the city in which their primary practice is located. In the naloxone provision questions (Appendix B) participants were asked to describe the weekly amount of naloxone dispensed before and during the pandemic. If there was a difference in dispensing, the participant was asked an open-ended question on what might explain the difference. Questions were asked related to barriers, facilitators, adjustments, and comfort level to naloxone provision in light of the COVID-19 pandemic. 

Survey responses that did not meet the eligibility criteria or that did not answer any naloxone provision questions were excluded from the analysis. The data was analyzed using descriptive statistics in Google Sheets. (Table S1 in Supplementary Data File and Analysis). Qualitative data from the open-ended responses were analyzed by thematic analysis using Google Sheets. The survey data underwent a sub-analysis, in which responses were compared as a function of pharmacy type or population size of the practice location. 

## 3. Results

### 3.1. Demographic Information among Survey Participants

A total of 408 responses were recorded. A total of 177 responses were removed because they failed the eligibility criteria, had no responses to questions about naloxone, or the response was invalid. This left 231 responses (completion rate = 56.6%) that were included in the analysis (Table 1). Almost all respondents were pharmacists (*n* = 229, 99.1%), while very few were pharmacy technicians (*n* = 2, 0.9%). While the sample of pharmacists was large, it was not large enough to be statistically representative of the 11,546 pharmacists in Ontario [18]. The sample of pharmacists would need to have been at least 372 in order to have a 95% confidence interval and a margin of error of 5%. The majority of respondents held a Bachelor of Pharmacy degree (*n* = 191, 82.7%). Some participants (*n* = 41, 17.7%) had five or fewer years of work experience, while almost half (*n* = 109, 47.2%) had 21 or more years of work experience. Approximately half of respondents were not from chain pharmacies (*n* = 108, 50.6%), and half were from chain pharmacies (*n* = 108, 46.8%). The remaining participants (*n* = 14, 2.6%) either practiced as relief pharmacists or did not indicate a practice site. 

### 3.2. Pharmacy Professionals’ Self-Reported Changes in Naloxone Dispensing

Approximately half of participants (*n* = 118, 51.1%) reported no change in their naloxone dispensing before and during the pandemic, while around a quarter (*n* = 53, 22.9%) of respondents indicated an increase in dispensing, and around another quarter (*n* = 57, 24.7%) indicated a decrease in dispensing (Table 2). For participants who worked in chain pharmacies and reported a change in dispensing, both increased (*n* = 23, 22.9%) and decreased (*n* = 30, 24.7%) dispensing were reported. Among participants in non-chain pharmacies reporting a change in dispensing, slightly more pharmacists reported an increase in dispensing (*n* = 30, 25.9%) vs. a decrease (*n* = 22, 19%). Participants working in small and large population centers who reported a change in dispensing were more likely to report a decrease than an increase. 

### 3.3. Reasons for Changes in Naloxone Dispensing

Participants who experienced a change in their naloxone dispensing were asked to provide a reason. Among those reporting a decrease, the most common reasons were a reduction in patient traffic in the pharmacy and COVID-19 measures preventing naloxone dispensing. One pharmacist explained how the reduction in foot traffic had affected their ability to dispense naloxone:

*“Being in less patient contact compared to before COVID, many people don’t want to wait for counselling on opioid medications, so we don’t get to recommend Naloxone kits as before.”*

Among those reporting an increase in naloxone dispensing, one reason was an increase in unregulated drug use among individuals. For example, some pharmacy professionals explained that because of the pandemic-induced isolation and associated mental health problems, individuals are consuming more opioids. One participant explained the situation as follows:

*“The isolation and job losses are creating more stressors. More people are looking to escape the pains of their circumstances. Excessive drug use has escalated, and syringe sales have also increased dramatically.”*

Another reason for an increase in dispensing was because of an increase in naloxone awareness. Not only were patients more aware of the importance of naloxone, but there was also promotion coming from pharmacies. One participant spoke of the increased demand by patients for naloxone kits:

*“More people are asking for them, and we are encouraging people to take more than one kit due to Fentanyl’s strong street presence.”*

### 3.4. Adjustments to Naloxone Dispensing

Over half of respondents reported that no adjustments have been made to dispensing naloxone in their practice during the COVID-19 pandemic (*n* = 137, 59.3%). For those that did change their dispensing, the top three adjustments to dispensing naloxone during the pandemic were training patients how to administer naloxone over video or phone (*n* = 56, 24.2%), offering to deliver naloxone kits (*n* = 42, 18.2%), and having the pharmacy technician offer naloxone at prescription intake (*n* = 30, 13.0%) (Table 3). When comparing adjustments made amongst population size and pharmacy type (Appendix C), over half of non-chain pharmacy participants (*n*= 72, 62.1%) reported feeling that no adjustments had been made during the pandemic, whereas a smaller portion of chain pharmacy participants (*n* = 61, 56.5%) felt this to be the case. The group with the highest proportion of participants reporting having a pharmacy technician offer naloxone at prescription intake were those located in the large population center (*n* = 16, 17%) when compared to the medium population (*n* = 7, 11.7%) and small population (*n* = 7, 9.3%) centers. 

### 3.5. Barriers and Facilitators to Naloxone Provision

The top three barriers to dispensing naloxone during the pandemic were related to a perceived decrease in access to or decreased demand by the public/patients. The top barrier was that patients were not asking for naloxone, followed by less face-to-face interaction with the public and finally reduced traffic inside the pharmacy (Table 4). When comparing barriers amongst population size and pharmacy type (Appendix C), two-thirds of participants in small population centers (*n* = 48, 64%) reported no-one asking for naloxone as the largest barrier, when compared to medium (*n* = 29, 48.3%) and large (*n* = 48, 51.1%) population centers. Participants in the large population centers (*n* = 14, 14.9%) or chain pharmacies (*n* = 17, 15.7%) reported that lack of time was a major barrier when compared to medium population centers (*n* = 6, 10%), small population centers (*n* = 6, 8%), and non-chain pharmacies (*n* = 8, 6.9%). Additionally, non-chain pharmacies reported less face-to-face interaction with the public (*n* = 69, 59.5%) and reduced traffic inside the pharmacy (*n* = 66, 56.9%) as barriers when compared to chain pharmacies (*n* = 53, 49.1%; *n* = 43, 39.8%). 

Many respondents reported feeling that nothing had helped them dispense naloxone during the COVID-19 pandemic. The top facilitator to dispensing naloxone during the pandemic was that patients were approaching pharmacists more due to medical office closures, followed by a lack of access to community social supports, and followed by patients being more concerned about being home alone (Table 5).

When comparing population size and pharmacy type with facilitators (Appendix C), it was found that the small population center had the highest proportion of participants who felt that nothing had helped them to dispense naloxone during the pandemic (*n* = 39, 52%), when compared to medium (*n* = 19, 31.7%) and large population centers (*n* = 39, 41.5%). When analyzing this sentiment by pharmacy type, far more non-chain pharmacy participants (*n* = 61, 52.6%) reported feeling that nothing had helped them during the pandemic compared to chain pharmacy participants (*n* = 36, 33.3%) that felt this to be the case. 

### 3.6. Comfort Level Dispensing Naloxone before and during the Pandemic

Comfort in dispensing naloxone remained unaffected by the pandemic (Table 6). Most participants felt very comfortable or comfortable before the pandemic (*n* = 202, 87.4%). During the pandemic, most participants were also very comfortable or comfortable (*n* = 196, 84.8%). No respondents reported feeling very uncomfortable before or during the pandemic.

### 3.7. What Made Pharmacy Professionals More Comfortable Dispensing Naloxone during the Pandemic?

Multiple participants (*n* = 76, 32.9%) reported that nothing had made them more comfortable dispensing naloxone during the pandemic, while fewer participants (*n*= 35, 15.1%) mentioned things that made them more comfortable. Many of these participants attributed an increase in their professional awareness of the importance of naloxone as making them more comfortable with naloxone provision. For example, some participants recognized that individuals were experiencing more stress and that there was an increase in opioid overdoses and deaths. One participant described the risk of patients being home alone as follows:

*“I feel I am more motivated to open a discussion about a patient being alone at home and the potential for overdose with prescription medications.”*

The implementation of COVID-19 measures also made participants more comfortable dispensing naloxone. For example, several participants mentioned having proper personal protective equipment (PPE), plexiglass, and a workplace that adhered to transmission prevention protocols as making them more comfortable with dispensing naloxone. Additionally, some participants mentioned continuing education on the topic of naloxone as making them more comfortable.

*“I’m currently taking the CAMH course for substance use disorder, and I think that has increased my comfort.”*

## 4. Discussion

Our results show that the sample of participants was divided into three groups with respect to naloxone dispensing: those who experienced no change, those who experienced an increase, and those who experienced a decrease. For those who experienced a decrease in naloxone dispensing, commonly reported reasons included less traffic in the pharmacy and COVID-19 measures (e.g., physical distancing). For those who experienced an increase, frequently mentioned reasons revolved around increased drug use as a result of the pandemic, patient awareness of naloxone, and pharmacy promotion of naloxone. Interestingly, more decreases than increases in dispensing were reported by participants practicing in chain pharmacies, while more increases than decreases in dispensing were reported for those practicing in non-chain pharmacies. Interestingly, previous studies have shown that chain pharmacies dispense or stock more naloxone than independent pharmacies [14,19,20,21,22,23]. The results of the present study may suggest that independent pharmacies are as able, or possibly better able, to manage the dispensing of naloxone in moments of crisis, like the COVID-19 pandemic. 

A large portion of pharmacists (55.4%) felt that no adjustments had been made to dispense naloxone during the pandemic, and this was also more common amongst non-chain pharmacies (62.1%) than chain pharmacies (56.5%). However, among the pharmacists reporting adjustments, the top three were training patients to administer naloxone over video or phone, followed by offering to deliver naloxone kits, and having the pharmacy technician offer naloxone at prescription intake. Future continuing education initiatives should encourage pharmacists, especially those who are not working in chain pharmacies, to adapt their practices for their patients who are isolating at home or would prefer not to come personally to the pharmacy. Outside of the context of the COVID-19 pandemic, pharmacists can make adjustments for their patients to support more naloxone distribution by offering to move to a separate space for counselling or to counsel about naloxone over the phone [24]. In addition, lessons learned during the current pandemic could serve as a basis for contingency planning for future major disruptions in naloxone access. A recent qualitative study of patients who consume opioids found that patients who are stigmatized the most do not ask for naloxone due to previous judgmental experiences in the environment of the pharmacy or with the pharmacist [24]. It is important for pharmacists to adapt their practice to create a comfortable space for their patients, provide positive reinforcement to their patients for getting a naloxone kit, and to recognize when they may be using stigmatizing language [24].

As this is the first survey to evaluate pharmacy professionals’ perceptions of the pandemic’s effect on naloxone distribution, many of the barriers and facilitators are unique. For example, the barriers of less face-to-face interaction with the public and reduced traffic inside the pharmacy are unique to this survey. However, the barrier of no-one asking for naloxone has been previously mentioned in other studies evaluating community pharmacy take home naloxone programs [14,25,26,27,28]. The national consensus guidelines for naloxone prescribing by pharmacists recommends that a naloxone kit be offered to every patient presenting with an opioid prescription [29]. The fact that most pharmacists are waiting for patients to ask for a naloxone kit suggests that continuing education is needed to teach pharmacists how to proactively offer naloxone and initiate conversations with their patients to overcome this barrier. Research has shown that patients want pharmacists to proactively offer naloxone with their opioid prescription as a way to minimize the stigma associated with naloxone [24]. It is possible that more education on how to proactively offer naloxone is needed for pharmacists working in small population centers, due to the fact that most reported no-one asking for naloxone as a barrier, when compared to the medium population and large population centers. In addition, non-chain pharmacies reported less face-to-face interaction with the public and reduced traffic in the pharmacy as being major barriers, while chain pharmacies did not report these barriers as often. Non-chain pharmacies may benefit from education on how to engage their patients, such as proactively calling patients to see if they would like naloxone when receiving an opioid prescription. Even before the pandemic, opioid overdose deaths were increasing significantly [5]. Studies have also been conducted to show that the provision of naloxone kits to opioid users is cost effective [30,31,32]. As the number of opioid overdoses appears to be increasing with the pandemic, it is imperative that pharmacy professionals be trained on the importance of being proactive in their approach to offering patients naloxone kits. 

The top three facilitators that were unique to this study included patients approaching pharmacists more due to medical office closures, lack of access to community social supports, and patients being more concerned about being home alone. This suggests that the ease in accessibility of pharmacies and pharmacists actively working on the frontlines during this pandemic have proven crucial for promoting harm reduction and promoting naloxone use. However, participants working in small population centers or non-chain pharmacies were more likely to report that nothing had helped them dispense naloxone during COVID-19. This suggests that there is room to investigate naloxone dispensing practices among independent and rural pharmacy practices to determine how to maximize naloxone dispensing in areas where the pharmacy may be the only access point for naloxone. This survey served as a good starting point to identify potential trends in naloxone kit provision by Ontario community pharmacy professionals. Future research should include quantitative measures of provincial naloxone distribution during the pandemic and a more in-depth qualitative study involving interviews with pharmacy professionals to develop a stronger understanding of why naloxone provision may have changed because of the pandemic and to identify more ways to increase proactively dispensing of naloxone. Although we noted several differences between the type of pharmacy and the population size, there were no apparent major predictors of changes in naloxone distribution based on pharmacy characteristics. 

A limitation to the study would be that 99.1% of the participants were pharmacists, but the survey intended to also capture pharmacy technicians. This means that the results reflect the views of mostly pharmacists. Moreover, the total number of pharmacists included in the survey (*n* = 229) was not large enough to have been statistically representative of Ontario’s 11,546 pharmacists [18]. To have had a 95% confidence interval with a 5% margin of error, a sample size of 372 pharmacists would have been required. Last, while this study demonstrated the potential for the existence of a relationship between pharmacy type and population size on naloxone dispensing, additional research is required to determine the nature of this relationship.

## 5. Conclusions

The effect of the pandemic on our sample of Ontario pharmacy professionals’ dispensing habits was divided into those who experienced change and those who experienced no change. Among those experiencing change, half reported an increase, while the other half reported a decrease. Our sub-analysis demonstrates that this difference could be caused by the participant’s type of pharmacy and the population size of their practice’s location. Participants in non-chain pharmacies saw more increases than decreases in naloxone dispensing, while the reverse was true for their chain pharmacy counterparts. This survey also showcased several adjustments being made to dispense naloxone during the pandemic, such as training patients via video or phone and delivering naloxone kits. Newly-identified barriers to naloxone provision included less face-to-face interaction with the public and reduced traffic inside the pharmacy, while newly-identified facilitators included patients approaching pharmacists more due to medical office closures and a lack of access to community social supports. This survey also identified that pharmacists continue to require training to proactively offer naloxone kits to their patients.

## Figures and Tables

**Table 1:** Demographics of survey participants.

Profession	Participants (%)
Pharmacy technician Pharmacist	2 (0.9%) 229 (99.1%)
**Education**	
Bachelor of Science (Pharmacy) BScPharm	191 (82.7%)
Doctor of Pharmacy (PharmD)	32 (13.9%)
Graduate degree	28 (12.1%)
Residency	10 (4.3%)
**Years in Practice**	
≤5	41 (17.7%)
6–10	35 (15.2%)
11–20	41 (17.7%)
≥21	109 (47.2%)
No response	5 (2.2%)
**Pharmacy type**	
Chain	108 (46.8%)
Independent	79 (34.2%)
Banner	38 (16.4%)
No response	1 (0.5%)
Other (relief, unspecified)	5 (2.1%)
**Population size**	
Population center with population < 1000	1 (0.4%)
Small population center (1000 ≤ population ≤ 29,999)	75 (32.5%)
Medium population center (30,000 ≤ population ≤ 99,999)	60 (26.0%)
Large population center (population ≥ 100,000)	94 (40.7%)
No response	1 (0.4%)

**Table 2:** Changes in naloxone dispensing practices.

	Overall Dispensing		
Increase	**53 (22.9%)**		
Equal	118 (51.1%)		
Decrease	57 (24.7%)		
Undetermined	3 (1.3%)		
**Total**	**231**		
	**Chain**	**Not Chain**	
Increase	**23 (22.9%)**	**30 (25.9%)**	
Equal	53 (51.1%)	63 (54.3%)	
Decrease	30 (24.7%)	22 (19.0%)	
Undetermined	2 (1.3%)	1 (0.8%)	
**Total**	**108**	**116**	
	**Small Population** **Center** (1000 ≤ population ≤ 29,999)	**Medium Population** **Center** (30,000 ≤ population ≤ 99,999)	**Large Population** **Center** (population ≥ 100,000)
Increase	12 (16.0%)	19 (31.7%)	**22 (23.4%)**
Equal	45 (60.0%)	28 (46.7%)	44 (46.8%)
Decrease	18 (24.0%)	12 (20%)	26 (27.7%)
Undetermined	0	1 (1.6%)	2 (2.1%)
Total	**75**	**60**	**94**

**Table 3:** Adjustments affecting naloxone dispensing during the pandemic

Adjustments	Participants (%)
No adaptations have been made	137 (59.3)
Training patients how to administer naloxone over video or phone	56 (24.2)
Offering to deliver naloxone kits	42 (18.2)
Having the pharmacy technician offer naloxone at prescription intake	30 (13.0)
Other	3 (1.3)

**Table 4:** Barriers affecting participant naloxone dispensing during the pandemic.

Barrier	Participants (%)
No-one asking for naloxone	127 (55.0)
Less face-to-face interaction with the public	125 (54.1)
Reduced traffic inside the pharmacy	112 (48.5)
Lack of time	26 (11.3)
No barriers	18 (7.8)
Other	13 (5.6)
Pharmacy not offering delivery	10 (4.3)
Difficulty stocking naloxone	9 (3.9)

**Table 5:** Facilitators affecting naloxone dispensing during the pandemic

Facilitator	Participants (%)
Nothing has helped me dispense naloxone during COVID-19	99 (42.9)
Patients approaching pharmacists more due to medical office closures	63 (27.3)
Lack of access to community social supports (e.g., public health nurses)	53 (23.0)
Patients are more concerned about being home alone	43 (18.6)
Patients more concerned about respiratory problems during the pandemic	42 (18.2)
Closure of supervised consumption sites	25 (10.8)
Other	11 (4.8)
No facilitator	16 (6.9)

**Table 6:** Participant comfort levels in dispensing naloxone before and during the pandemic.

Facilitator	Before	During
Very comfortable	128 (55.4%)	117 (50.6%)
Comfortable	74 (32%)	79 (34.2%)
Neutral	19 (8.2%)	27 (11.7%)
Uncomfortable	6 (2.6%)	5 (2.2%)
Very uncomfortable	0	0
No response	4 (1.7%)	3 (1.3%)

## Data Availability

The data presented in this study are available in the supplementary data set.

## Supplementary Materials

The following are available online at https://www.mdpi.com/article/10.3390/pharmacy9030129/s1, Table S1: Supplementary Data File and Analysis.

## Appendix A

### Appendix A.1. Eligibility Questions

Eligibility Questions (2)

1. Are you a pharmacy technician or Part A pharmacist registered with the Ontario College of Pharmacists?
  - Yes
  - No (if no, end survey and thank participant but explain ineligibility)
2. Have you been practicing as a pharmacist or pharmacy technician in the community pharmacy setting for the last year?
  Please note, ‘community pharmacy’ refers to corporate, franchise, banner, or independently owned pharmacies
  - Yes
  - No (if no, end survey and thank participant but explain ineligibility)

## Appendix B

### Appendix B.1. Survey Questionnaire

#### Appendix B.1.1. Demographic Questions (5)

1. What type of pharmacy professional are you?
  - Pharmacist
  - Pharmacy technician
2. Education (SELECT ALL THAT APPLY)
  - BScPharm
  - PharmD
  - Hospital Residency
  - Graduate Degree
  - Pharmacy technician training
3. Years in Practice
  (open text)
4. Type of Community Pharmacy
  If you work at multiple types of community pharmacies, please indicate the type you work at most frequently.
  - Chain
  - Independent
  - Banner
  - Other
5. Describe the population size of the area in which your community pharmacy practice is located.
  If you work at multiple community pharmacies, please answer the question according to the community pharmacy you work at most frequently.
  - Population center with population less than 1,000
  - Small population center (population between 1,000 and 29,999)
  - Medium population center (population between 30,000 and 99,999)
  - Large population center (population 100,000 and over)

#### Appendix B.1.2. Survey Questions (13)

1. How many naloxone kits ***per week*** did you dispense, on average, **before** the start of the COVID-19 pandemic?
  *[Free-text field]*
2. How many naloxone kits ***per week*** do you dispense, on average, **now** with the COVID-19 pandemic?
  *[Free-text field]*
3. If the number of naloxone kits dispensed now versus before the pandemic is different, what do you think is impacting an increase or decrease in your naloxone dispensing practices?
  *[Free-text field]*
4. Please share any *barriers* you have found to dispensing naloxone since the beginning of the COVID-19 pandemic. (SELECT ALL THAT APPLY)
  - Less face-to-face interaction with the public
  - Reduced traffic inside the pharmacy
  - Pharmacy not offering delivery
  - Difficulty stocking naloxone
  - No-one asking for naloxone
  - Lack of time
  - Other: [Free text]
5. Please share what has facilitated naloxone dispensing since the beginning of the COVID-19 pandemic (SELECT ALL THAT APPLY).
  - Patients more concerned about respiratory problems during the pandemic
  - Patients are more concerned about being home alone
  - Closure of supervised consumption sites
  - Lack of access to community social supports (e.g., public health nurses)
  - Patients approaching pharmacists more due to medical office closures
  - Nothing has helped me dispense naloxone during COVID-19
  - Other: [Free-text field]
6. What adaptations (if any) have you made in your daily practice to support the dispensing of naloxone during the COVID-19 pandemic? (SELECT ALL THAT APPLY)
  - Offering to deliver naloxone kits
  - Training patients how to administer naloxone over video or phone
  - Having the pharmacy technician offer naloxone at prescription intake
  - No adaptations have been made
  - Other [Free-text field]
7. How would you rate your comfort in dispensing naloxone *before* the COVID-19 pandemic?
  - Very comfortable
  - Comfortable
  - Neutral
  - Uncomfortable
  - Very uncomfortable
8. How would you rate your comfort in dispensing naloxone *during* the COVID-19 pandemic?
  - Very comfortable
  - Comfortable
  - Neutral
  - Uncomfortable
  - Very uncomfortable
9. Has there been anything that has made you more comfortable dispensing naloxone during the pandemic?
  *[Free-text field]*
10. Have you ever had to administer naloxone inside the pharmacy?
  - Yes
  - No
11. When you administered naloxone, was it:
  - Before the COVID-19 pandemic
  - During the COVID-19 pandemic
  - Both before and during the COVID-19 pandemic
    *(Qualtrics set up such that if question 10 answered with “No”, then this question is skipped)*
12. During one of your shifts, has another pharmacy staff member ever had to administer naloxone inside the pharmacy?
  - Before the COVID-19 pandemic
  - During the COVID-19 pandemic
  - Both before and during the COVID-19 pandemic
    *(Qualtrics set up such that if question 12 answered with “No”, then this question is skipped)*
13. When they administered naloxone, was it:
  - Yes
  - No

## Appendix C

**Table A1:** Comparing barriers, facilitators, and adjustments among population size and pharmacy type.

	Population Size (%)			Pharmacy Type (%)	
Barriers	Small Population Center (1000 ≤ Population ≤ 29,999)	Medium Population Center (30,000 ≤ Population ≤ 99,999)	Large Population Center (population ≥ 100,000)	Chain	Not Chain
No-one asking for naloxone	48 (64)	29 (48.3)	48 (51.1)	62 (57.4)	61 (52.6)
Less face-to-face interaction with the public	42 (56)	35 (58.3)	47 (50)	53 (49.1)	69 (59.5)
Reduced traffic inside the pharmacy	36 (48)	29 (48.3)	47 (50)	43 (39.8)	66 (56.9)
Lack of time	6 (8)	6 (10)	14 (14.9)	17 (15.7)	8 (6.9)
No barriers	5 (6.7)	6 (10)	7 (7.4)	8 (7.4)	10 (8.6)
Pharmacy not offering delivery	1 (1.3)	5 (8.3)	4 (4.3)	6 (5.6)	4 (3.4)
Difficulty stocking naloxone	1 (1.3)	3 (5)	5 (5.3)	6 (5.6)	3 (2.6)
Other	4 (5.3)	6 (10)	3 (3.2)	6 (5.6)	7 (6.0)
**Facilitators**					
Nothing has helped dispense naloxone during COVID-19	39 (52)	19 (31.7)	39 (41.5)	36 (33.3)	61 (52.6)
Patients approaching pharmacists more due to medical office closures	20 (26.7)	18 (30)	25 (26.6)	36 (33.3)	26 (22.4)
Lack of access to community social supports (e.g., public health nurses)	17 (22.7)	16 (26.7)	20 (21.3)	30 (27.8)	21 (18.1)
Patients are more concerned about being home alone	9 (12)	15 (25)	19 (20.2)	20 (18.5)	23 (19.8)
Patients are more concerned about respiratory problems during the pandemic	11 (14.7)	12 (20)	19 (20.2)	16 (14.8)	25 (21.6)
Closure of supervised consumptions sites	6 (8)	8 (13.3)	11 (11.7)	12 (11.1)	11 (9.5)
Other	3 (4)	2 (3.3)	6 (6.4)	7 (6.5)	4 (3.4)
No facilitator	2 (2.7)	4 (6.7)	10 (10.6)	8 (7.4)	8 (6.9)
Adjustments					
No adjustments have been made	48 (64)	33 (55)	54 (57.4)	61 (56.5)	72 (62.1)
Training patients how to administer naloxone over video or phone	17 (22.7)	17 (28.3)	22 (23.4)	28 (25.9)	27 (23.3)
Offering to deliver naloxone kits	11 (14.7)	13 (21.7)	18 (19.1)	19 (17.6)	21 (18.1)
Having the pharmacy technician offer naloxone at prescription intake	7 (9.3)	7 (11.7)	16 (17)	15 (13.9)	15 (12.9)
Other	1 (1.3)	1 (1.7)	1 (1.1)	0 (0)	3 (2.6)
